# Association between the neutrophil-to-albumin ratio and mortality in patients undergoing tracheal intubation: a retrospective cohort study

**DOI:** 10.3389/fmed.2026.1804074

**Published:** 2026-04-01

**Authors:** Xiaoping Huang, Junfan Chen, Weide Lin, Bixia Lin

**Affiliations:** 1 Department of Anesthesiology, The First Hospital of Putian City, Putian, China; 2 Department of Medical Equipment, The First Hospital of Putian City, Putian, China; 3 Department of Ultrasonography, The First Hospital of Putian City, Putian, China

**Keywords:** intensive care unit, mechanical ventilation, mortality, neutrophil-to-albumin ratio, retrospective cohort study

## Abstract

**Introduction:**

Tracheal intubation in intensive care unit (ICU) patients is associated with significant risks, necessitating simple mortality predictors. We investigated the relationship between neutrophil percentage-to-albumin ratio (NPAR) and in-hospital and ICU mortality in intubated patients.

**Methods:**

This retrospective cohort study analyzed 1,401 intubated ICU patients from MIMIC-IV. Multivariable Cox regression and smooth curve fitting were used to assess the association between NPAR and mortality. A two-stage linear regression model and threshold analysis evaluated nonlinear relationships.

**Results:**

Among patients (mean age 62.7 ± 17.1 years; 43.6% female), NPAR exhibited a U-shaped relationship with mortality, with an inflection point at 2.2 observed in this cohort. Below this inflection point, hazard ratios (HRs) were 0.656 (95% CI: 0.492–0.875) for in-hospital mortality and 0.586 (95% CI: 0.413–0.831) for ICU mortality. Above 2.2, HRs increased to 1.187 (95% CI: 1.097–1.286) and 1.15 (95% CI: 1.047–1.264), respectively.

**Conclusion:**

NPAR demonstrates a U-shaped association with mortality in intubated ICU patients, with elevated risks above 2.2. Further large-scale studies are needed to validate these findings.

## 1 Introduction

Tracheal intubation and mechanical ventilation are vital interventions for airway management across clinical settings (1). These procedures provide crucial respiratory support during surgery, intensive care, and perioperative stabilization of critically ill patients (1). Endotracheal intubation is the gold standard for airway management (2, 3), with 10%–15% of intensive care unit (ICU) patients worldwide requiring this procedure annually (4, 5). In the U.S., over 1.5 million patients require endotracheal intubation annually, with increasing prevalence (6). This procedure primarily maintains airway patency and safety (7), while also facilitating secretion clearance and supporting diagnostic/therapeutic interventions. Although endotracheal intubation plays a critical role in clinical treatment, related procedures (such as repeated intubation attempts in difficult airways, intubation duration, and changes in posture during intubation) may increase the risk of airway injury (8, 9), while subsequent mechanical ventilation management may lead to complications such as oxygen toxicity, acquired muscle weakness, and ventilator-induced lung injury (10). Therefore, identifying easily accessible and low-cost prognostic indicators is crucial for improving outcomes in intubated ICU patients. This helps to reduce the burden on patients and lower the mortality rate.

Scoring systems such as SOFA and APACHE II are widely used in intensive care, but their reliance on complex parameters limits their application value in scenarios requiring rapid decision-making for endotracheal intubation. The neutrophil percentage-to-albumin ratio (NPAR) is an easily obtainable and cost-effective biomarker (11), which may provide a more efficient auxiliary tool for intubation decision-making. As an inflammation-based prognostic predictor (12), NPAR has been associated with mortality risk in various diseases, including chronic obstructive pulmonary disease (COPD), stroke, coronary artery disease, sepsis, acute kidney injury, and peritoneal dialysis patients (13–18). In addition, previous studies have found that NPAR has significant clinical value in assessing the prognosis of elderly patients with hip fractures 1 year after surgery (19). NPAR is not only capable of predicting the occurrence of stroke-associated infection (20), but it is also an independent prognostic factor for ischemic stroke patients admitted to the ICU (21). The prognostic value of NPAR stems from the pathophysiological interplay between neutrophils and albumin. Lower neutrophil percentages reflect a moderated inflammatory response, reducing the risk of excessive inflammation-induced tissue damage (22). Conversely, higher albumin levels help maintain endothelial integrity, scavenge oxygen free radicals, mitigate oxidative stress, and regulate the balance between pro- and anti-inflammatory factors (23). This dual regulatory mechanism involving both inflammatory response and nutritional status may constitute the potential biological basis for the association between NPAR and clinical outcomes. Despite NPAR’s established prognostic value in various diseases, its association with outcomes in intubated ICU patients remains unexplored. Using the Medical Information Marketplace in Intensive Care IV (MIMIC-IV) database, we investigated NPAR’s relationship with in-hospital and ICU mortality in this population, findings that could inform clinical decision-making.

## 2 Materials and methods

### 2.1 Database introduction

Our study data was extracted from the open-source MIMIC-IV clinical database. The MIMIC-IV database contains a comprehensive dataset of 50,920 patients admitted to the ICU at Beth Israel Deaconess Medical Center from 2008 to 2019 (24), including demographic baseline characteristics, vital signs, laboratory tests, medication records, and diagnoses coded according to the International Classification of Diseases, Ninth Revision (ICD-9) and Tenth Revision (ICD-10). After successfully completing the online check and executing the data usage agreement, the principal investigator Weide Lin obtained access to the database (certification number: 62407435). The study adheres to the ethical guidelines of the Declaration of Helsinki. All datasets used in this study can be freely accessed in the MIMIC-IV v2.2 database^1^. The establishment of the MIMIC-IV database was approved by the Institutional Review Board of Beth Israel Deaconess Medical Center and its affiliated institution, the Massachusetts Institute of Technology. Due to the anonymization of the data, informed consent was not sought.

### 2.2 Patients

The MIMIC-IV database includes a total of 73,181 patients, of which there are 4,012 intubated patients admitted to the ICU who are over 18 years old. We excluded 1,991 patients with missing albumin data and 459 patients with missing neutrophil percentage data, resulting in a final study population of 1,562 intubated patients. Subsequently, we excluded 149 patients with ICU stay less than 24 h and 12 patients with hospital stay less than 24 h, ultimately including 1,401 intubated patients. We only include the data for the patient’s first ICU admission and the corresponding hospital stay (25).

### 2.3 Variable extraction and outcome

We extracted data including demographics, vital signs, and laboratory tests using PostgreSQL version 13.9. The selection of covariates was based on variables previously established in the literature as being associated with mortality and the biological plausibility of confounding effects. All laboratory indicators and clinical variables were collected after admission to the ICU, and tracheal intubation procedures were performed during the ICU stay. Covariates included in our analysis were age, gender, race, heart rate, systolic blood pressure (SBP), respiratory rate (Resp), oxygen saturation (SpO_2_), glucose, prothrombin time (PT), international normalized ratio (INR), sodium (Na), potassium (K), calcium (Ca), chlorine (Cl), acute kidney injury stage (AKI stage), myocardial infarction (MI), chronic obstructive pulmonary disease (COPD), cerebrovascular disease (CVD), peripheral vascular disease (PVD), sepsis, mechanical ventilation (MV), elective surgery, hospital mortality, and intensive care unit mortality (ICU mortality). NPAR was calculated using the following formula: NPAR = neutrophil percentage (%) × 100/albumin concentration (g/dL) (26). The neutrophil percentage and albumin concentration were both determined using the first measured values after ICU admission. If multiple records existed at the same time point, the earliest one was selected for analysis. Additionally, patients were grouped according to NPAR, with the primary outcome being in-hospital mortality and the secondary outcome being ICU mortality.

### 2.4 Statistical analysis

Patients were divided into three groups based on trichotomous NPAR levels (NPAR <2.41, 2.41 ≤ NPAR <3.38, NPAR ≥3.38).

Categorical variables are expressed as proportions (%) and were tested with a chi-square test or Fisher’s exact test. Continuous variables with a normal distribution are depicted as the mean ± standard deviation (SD) and were compared using Student’s t test or one-way ANOVA. For continuous variables not conforming to a normal distribution, the data are shown as the median and interquartile range (IQR), and the Kruskal–Wallis H test was used for statistical analysis. Since all covariates had low missing rates (range: 0.57%–2.28%), this study employed complete-case analysis for the statistical analysis.

Multivariable Cox regression analyses were adopted to assess the independent association between NPAR levels and the risk of in-hospital mortality and ICU mortality. The model adjusted for different covariates uses the extended Cox proportional hazards model. Prior to modeling, variance inflation factors (VIF) were calculated for all covariates, with all values <5, indicating no substantial multicollinearity. The models are as follows: The crude model was unadjusted. Model 1 was adjusted for sex and age. Model 2, in addition to the variables in Model 1, further adjusted for covariates with an effect size change greater than 10%, including MI, COPD, CVD, PVD, and sepsis. Model 3, on top of Model 2, adjusted for covariates with a univariate analysis *p*-value < 0.1, including race, heart rate, systolic blood pressure, Resp., SpO_2_, glucose, PT, INR, Na, K, Cl, and AKI stage. Model 4, beyond Model 3, adjusted for covariates reported as clinically significant in previous studies, additionally incorporating Ca, MV, and elective surgery.

We employed a two-piecewise regression model and smooth curve analysis to explore the nonlinear relationship between NPAR levels and in-hospital as well as ICU mortality. The inflection point was determined using the maximum likelihood method. To compare the fit of the models, we employed the likelihood ratio test to evaluate the differences between the single-variable regression model and the two-piecewise regression model. Furthermore, we conducted threshold effect analysis to assess the potential of NPAR levels in predicting in-hospital and ICU mortality. The identified threshold of 2.2 is data-driven and exploratory, and therefore requires external validation before clinical application.

Furthermore, to determine whether the relationship between NPAR levels and patients with tracheal intubation in the ICU was stable across populations, interaction and subgroup analyses were performed according to age (<65, or ≥65 years), gender (female or male), MI (yes or no), COPD (yes or no), sepsis (yes or no) and MV (yes or no). In the multivariable Cox regression model 4, adjustments are made for all stratified factors, and interactions between subgroups are analyzed using the likelihood ratio test. To evaluate the incremental predictive value of NPAR over the SOFA score, we calculated the net reclassification improvement (NRI) and integrated discrimination improvement (IDI). The baseline model included the SOFA score alone, and the new model added NPAR.

All analyses were performed using the statistical software package R. version 4.4.2 (R Foundation for Statistical Computing, Vienna, Austria) and Free Statistics software version 1.9.2 (27). *p* values < 0.05 (two-sided) were considered statistically significant.

### 2.5 Sensitivity analysis

In the sensitivity analysis, we categorized the primary diagnoses of intubated patients into six groups (neurological diseases, respiratory diseases, cardiovascular diseases, digestive diseases, infectious diseases, and others) and included the primary diagnosis as a covariate in the Cox regression model, while conducting subgroup analyses based on primary diagnoses to assess the stability of the results. Additionally, we adjusted for clinically important covariates including mechanical ventilation duration (missing rate 1.3%), acute respiratory distress syndrome (missing rate 30.2%), creatinine, glucocorticoid use, and albumin dosage. For variables with missing data such as mechanical ventilation duration and ARDS, we employed multiple imputation to generate five imputed datasets and selected one of the imputed datasets for analysis to ensure the reliability of the findings.

## 3 Results

### 3.1 Population of the study

**Figure 1:**
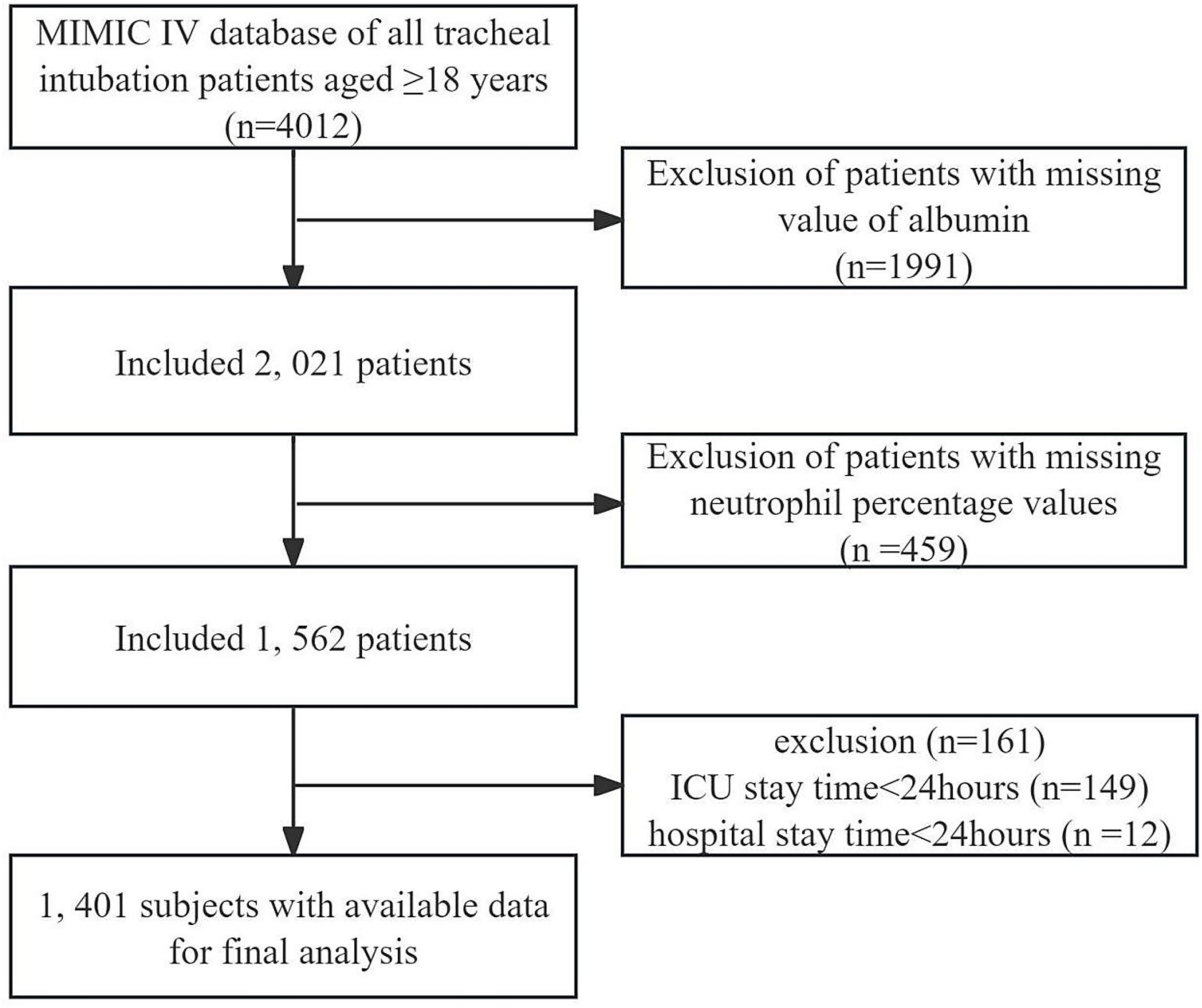
Schematic representation of the participant selection process and distribution of participant groups.

Based on the above inclusion and exclusion criteria, 1,401 patients were identified in the final cohort. The flow chart of the study patients is presented in Figure 1.

### 3.2 Participants and demographic characteristics

The baseline characteristics of patients are presented in Table 1. This cohort’s in-hospital mortality rate is 28.41%; the ICU mortality rate is 20.84%. The patient age was 62.7 ± 17.1 years, and approximately 56.4% patients were men, most of whom were white. Patients enrolled were categorized into three groups based on the tertiles of NPAR: NPAR <2.41 was considered as the lower group, 2.41 ≤ NPAR < 3.38 was considered as the middle group, and ≥3.38 was considered as the higher group. Compared to the lower NPAR group, patients in the higher NPAR group had faster heart and respiratory rates, higher PT and INR levels, higher in-hospital and ICU mortality, were more prone to sepsis, less likely to develop CVD, and had lower SBP.

**Table 1:** Baseline characteristics of participants.

Variables	Total (*n* = 1,401)	NPAR <2.41 (*n* = 465)	2.41 ≤ NPAR <3.38 (*n* = 471)	NPAR ≥3.38 (*n* = 465)	*p*-value
Age (years)	62.7 ± 17.1	61.3 ± 17.6	64.5 ± 17.0	62.1 ± 16.6	0.01
Gender, *n* (%)					0.033
Female	611 (43.6)	182 (39.1)	224 (47.6)	205 (44.1)	
Male	790 (56.4)	283 (60.9)	247 (52.4)	260 (55.9)	
Race/ethnicity, *n* (%)					0.177
White	874 (62.4)	280 (60.2)	293 (62.2)	301 (64.7)	
African Americans	148 (10.6)	57 (12.3)	55 (11.7)	36 (7.7)	
Other	379 (27.1)	128 (27.5)	123 (26.1)	128 (27.5)	
Heart rate (bpm)	112.6 ± 22.9	110.2 ± 21.1	111.5 ± 23.9	116.2 ± 23.2	<0.001
SBP (mmHg)	151.6 ± 25.2	154.4 ± 25.8	151.4 ± 25.0	149.0 ± 24.3	0.004
Resp (bpm)	29.2 ± 6.9	28.5 ± 6.6	29.1 ± 6.9	30.1 ± 7.0	0.002
SpO_2_ (%)	99.7 ± 0.9	99.7 ± 0.8	99.6 ± 0.9	99.7 ± 1.1	0.297
Glucose (mmol/L)	8.8 (6.9, 12.4)	8.4 (6.8, 12.5)	8.7 (6.9, 11.7)	9.1 (7.1, 13.1)	0.076
PT (s)	15.1 (13.2, 20.1)	13.8 (12.4, 17.8)	14.7 (13.3, 18.6)	16.3 (13.9, 23.8)	<0.001
INR (s)	1.4 (1.2, 1.8)	1.2 (1.1, 1.6)	1.3 (1.2, 1.7)	1.5 (1.2, 2.2)	<0.001
Na (mmol/L)	140.3 ± 6.1	140.5 ± 5.9	140.3 ± 6.1	140.0 ± 6.3	0.486
K (mmol/L)	4.8 ± 1.0	4.7 ± 1.0	4.8 ± 1.0	4.8 ± 0.9	0.389
Ca (mmol/L)	2.1 ± 0.3	2.2 ± 0.3	2.1 ± 0.3	2.1 ± 0.4	<0.001
Cl (mmol/L)	107.3 ± 7.5	106.6 ± 7.3	106.9 ± 7.5	108.3 ± 7.6	<0.001
AKI stage					<0.001
0	701 (50.0)	270 (58.1)	223 (47.3)	208 (44.7)	
1	378 (27.0)	122 (26.2)	139 (29.5)	117 (25.2)	
2	145 (10.3)	35 (7.5)	54 (11.5)	56 (12)	
3	177 (12.6)	38 (8.2)	55 (11.7)	84 (18.1)	
MI, *n* (%)					0.144
No	1,214 (86.7)	414 (89)	399 (84.7)	401 (86.2)	
Yes	187 (13.3)	51 (11)	72 (15.3)	64 (13.8)	
COPD, *n* (%)					0.008
No	1,019 (72.7)	351 (75.5)	318 (67.5)	350 (75.3)	
Yes	382 (27.3)	114 (24.5)	153 (32.5)	115 (24.7)	
CVD, *n* (%)					0.004
No	1,167 (83.3)	366 (78.7)	400 (84.9)	401 (86.2)	
Yes	234 (16.7)	99 (21.3)	71 (15.1)	64 (13.8)	
PVD, *n* (%)					0.259
No	1,257 (89.7)	426 (91.6)	418 (88.7)	413 (88.8)	
Yes	144 (10.3)	39 (8.4)	53 (11.3)	52 (11.2)	
Sepsis, *n* (%)					<0.001
No	237 (16.9)	127 (27.3)	58 (12.3)	52 (11.2)	
Yes	1,164 (83.1)	338 (72.7)	413 (87.7)	413 (88.8)	
MV, *n* (%)					0.616
No	357 (25.5)	114 (24.5)	117 (24.8)	126 (27.1)	
Yes	1,044 (74.5)	351 (75.5)	354 (75.2)	339 (72.9)	
Elective surgery, *n* (%)					0.716
No	1,394 (99.5)	464 (99.8)	468 (99.4)	462 (99.4)	
Yes	7 (0.5)	1 (0.2)	3 (0.6)	3 (0.6)	

Continuous variables are presented as mean ± SD or median (quartile), while categorical variables are presented as absolute numbers (percentages).

SBP, systolic blood pressure; Resp., respiratory; SpO_2,_ pulse oximetry derived oxygen saturation; PT, prothrombin time; INR, international normalized ratio; Na, sodium; K, potassium; Ca, calcium; Cl, chlorine; AKI stage, acute kidney injury stage; MI, myocardial infarction; COPD, chronic obstructive pulmonary disease; CVD, cerebrovascular disease; PVD, peripheral vascular disease; MV, mechanical ventilation; ICU mortality, intensive care unit mortality.

### 3.3 Results of cox regression analysis

The univariate analysis of in-hospital mortality and ICU mortality for patients with tracheal intubation in the ICU is shown in Supplementary Table S1. It indicated that age, Heart rate, SBP, Resp., SpO_2_, Glucose, PT, INR, Na, K, Cl, AKI stage, MI and NPAR are risk factors for in-hospital mortality (*p* < 0.05); age, Heart rate, SBP, Resp., SpO_2_, Glucose, PT, INR, K, Cl, AKI stage, MI and NPAR are risk factors for ICU mortality (*p* < 0.05). The K–M curves show a significant correlation between NPAR and the risk of in-hospital mortality as well as ICU mortality (Supplementary Figure S1).

Table 2 presents the results of multivariable Cox regression analysis for the association between NPAR and mortality. When analyzed as a continuous variable, NPAR showed a significant positive association with in-hospital and ICU mortality in the crude model (HR range 1.04–1.05, all *p* < 0.05). However, this association was no longer significant in Models 3 and 4 after full adjustment. Nevertheless, when considered as a categorical variable, with the second group of NPAR (2.41 ≤ NPAR < 3.38) as the baseline reference, higher NPAR levels (NPAR ≥ 3.38) were associated with a higher risk of in-hospital mortality and ICU mortality (Crude model, HR range 1.58–1.59, *p* < 0.05 for all). The hazard ratios for NPAR levels were significant in all four models. According to the fully adjusted model 4, patients with higher NPAR levels (NPAR ≥ 3.38) had a 61% increased risk of in-hospital mortality and a 62% increased risk of ICU mortality compared to the reference group of NPAR (2.41 ≤ NPAR < 3.38) (Model IV, HR range 1.61–1.62, *p* < 0.05 for all).

**Table 2:** Relationships between NPAR, in-hospital mortality, and ICU mortality in different models.

Variable	Crude model		Model I		Model II		Model III		Model IV	
	HR (95% CI)	*p-*value	HR (95% CI)	*p-*value	HR (95% CI)	*P-*value	HR (95% CI)	*p-*value	HR (95% CI)	*p-*value
Hospital mortality										
NPAR as continuous	1.05 (1.02 ~ 1.07)	0.001	1.05 (1.02 ~ 1.08)	<0.001	1.05 (1.02 ~ 1.08)	<0.001	1.01 (0.99 ~ 1.04)	0.324	1.02 (0.99 ~ 1.04)	0.31
2.41 ≤ NPAR <3.38	Ref		Ref		Ref		Ref		Ref	
NPAR <2.41	0.92 (0.71 ~ 1.19)	0.524	1.02 (0.78 ~ 1.32)	0.897	1.03 (0.79 ~ 1.35)	0.804	1.14 (0.87 ~ 1.49)	0.331	1.11 (0.85 ~ 1.45)	0.457
NPAR ≥3.38	1.58 (1.25 ~ 1.99)	<0.001	1.7 (1.34 ~ 2.15)	<0.001	1.69 (1.33 ~ 2.14)	<0.001	1.6 (1.25 ~ 2.05)	<0.001	1.61 (1.26 ~ 2.07)	<0.001
P for trend		<0.001		<0.001		<0.001		0.006		0.003
ICU mortality										
NPAR as continuous	1.04 (1 ~ 1.07)	0.025	1.04 (1.01 ~ 1.08)	0.012	1.04 (1.01 ~ 1.08)	0.021	1 (0.96 ~ 1.04)	0.933	1 (0.96 ~ 1.04)	0.915
2.41 ≤ NPAR <3.38	Ref		Ref		Ref		Ref		Ref	
NPAR <2.41	0.95 (0.7 ~ 1.29)	0.738	1.04 (0.76 ~ 1.41)	0.81	1.08 (0.79 ~ 1.47)	0.642	1.21 (0.89 ~ 1.66)	0.226	1.18 (0.86 ~ 1.61)	0.306
NPAR ≥3.38	1.59 (1.21 ~ 2.09)	0.001	1.7 (1.29 ~ 2.24)	<0.001	1.7 (1.29 ~ 2.24)	<0.001	1.6 (1.2 ~ 2.14)	0.002	1.62 (1.21 ~ 2.17)	0.001
P for trend		<0.001		<0.001		0.001		0.047		0.025

Crude model was not adjusted.

Model 1 was adjusted for gender + age.

Model 2 was adjusted for model 1 + MI + COPD + CVD + PVD + sepsis.

Model 3 was adjusted for model 2 + race + heart rate + SBP + Resp + SpO_2_ + glucose + PT + INR + Na + K + Cl + AKI stage.

Model 4 was adjusted for model 3 + Ca + MV + Elective surgery.

MI, myocardial infarction; COPD, chronic obstructive pulmonary disease; CVD, cerebrovascular disease; PVD, peripheral vascular disease; SBP, systolic blood pressure; Resp., respiratory; SpO_2,_ pulse oximetry derived oxygen saturation; PT, prothrombin time; INR, international normalized ratio; Na, sodium; K, potassium; Cl, chlorine; AKI stage, acute kidney injury stage; Ca, calcium; MV, mechanical ventilation.

### 3.4 Nonlinear relationship between NPAR levels and in-hospital mortality and ICU mortality

**Figure 2:**
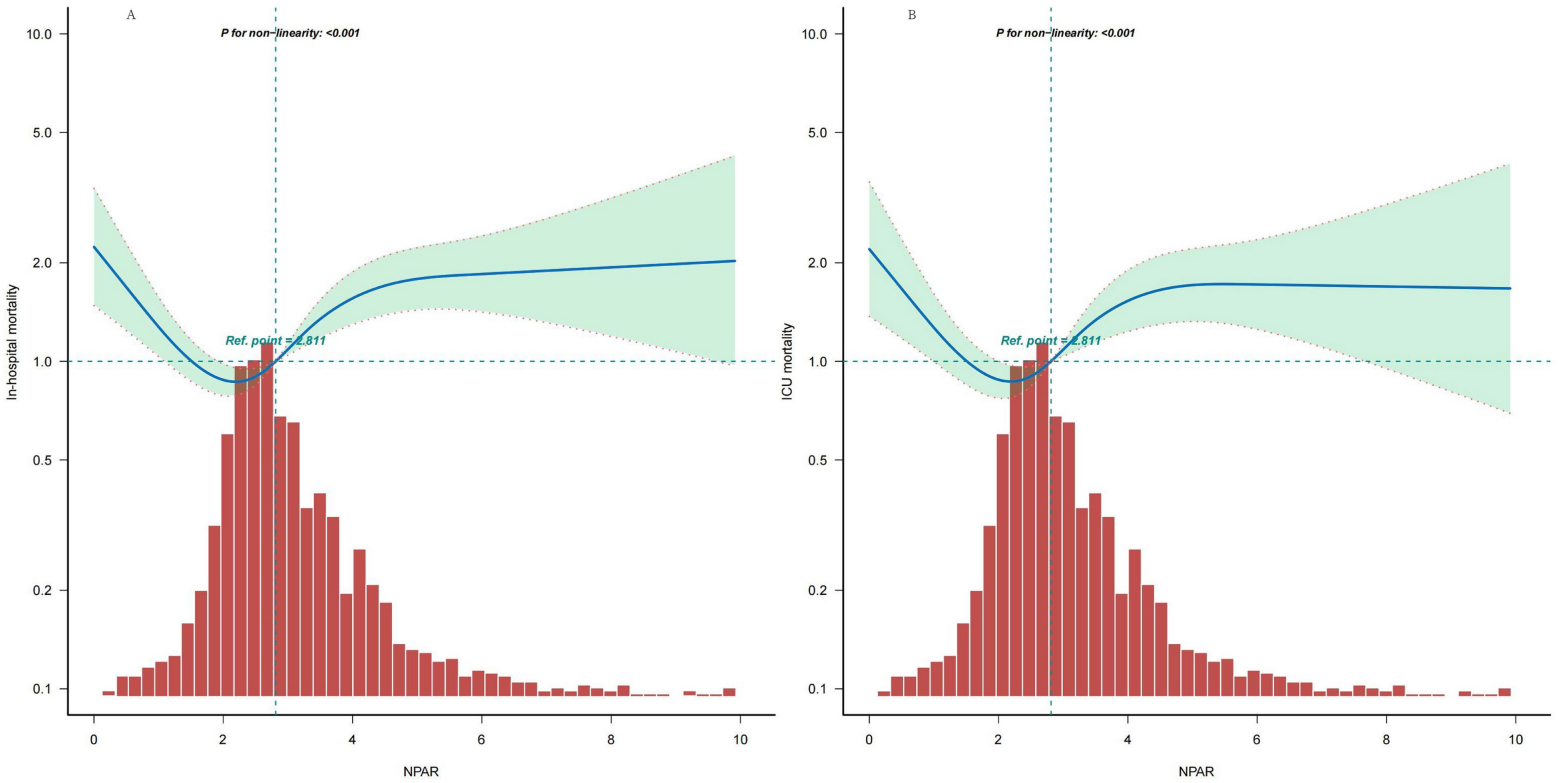
**(A)** Nonlinear dose–response relationship between NPAR and in-hospital mortality. **(B)** Nonlinear dose–response relationship between NPAR and ICU mortality. The analysis was adjusted for age, gender, race, heart rate, SBP, Resp., SpO_2_, glucose, PT, INR, Na, K, Ca, Cl, AKI stage, MI, COPD, CVD, PVD, sepsis, MV, and elective surgery. The blue line represents the estimated values, and the green area indicates the corresponding 95% confidence intervals. Only 99% of the data is displayed.

Multivariable Cox regression and smooth curve analysis reveal a nonlinear relationship between NPAR levels and the risks of in-hospital and ICU mortality (Figure 2). The piecewise linear regression model indicates that the NPAR threshold is 2.2 (Table 3), beyond which there is a significant increase in the risk of in-hospital and ICU mortality (HR from 1.15 to 1.187, *p* < 0.05, Table 3 and Figure 2). For each one-unit increase in NPAR, the risk of in-hospital mortality increases by 18.7%, and the risk of ICU mortality increases by 15%. Below the threshold, the risk of in-hospital mortality and ICU mortality decreased rapidly (HR range 0.586–0.656, *p* < 0.05 for all, Table 3 and Figure 2), for every one-unit decrease in NPAR level, the risk of in-hospital and ICU mortality decreases by 34.4% and 41.4%, respectively.

**Table 3:** Threshold effect analysis of NPAR levels on in-hospital mortality and ICU mortality.

Hospital mortality				ICU mortality			
Threshold of driving pressure	HR	95% CI	*p*-value	Threshold of driving pressure	HR	95% CI	*p*-value
NPAR <2.2	0.656	0.492, 0.875	0.0042	NPAR <2.2	0.586	0.413, 0.831	0.0027
NPAR ≥2.2	1.187	1.097, 1.286	<0.001	NPAR ≥2.2	1.15	1.047, 1.264	0.0036
Likelihood ratio test			<0.001	Likelihood ratio test			<0.001

HR, hazard ratio, CI, confidence interval. Both adjustment factors included age, gender, race, heart rate, SBP, Resp., SpO_2,_ glucose, PT, INR, Na, K, Ca, Cl, AKI stage, MI, COPD, CVD, PVD, sepsis, MV, Elective surgery. Only 99% of the data is displayed.

### 3.5 Subgroup analyses and interaction effects

We conducted stratified analysis and interaction analysis to assess whether the association between NPAR levels and in-hospital mortality and ICU mortality exists in different subgroups. After stratification by age, gender, MI, COPD, sepsis and MV, we found significant interactions between NPAR levels and both in-hospital mortality and ICU mortality in the presence or absence of MV groups. No significant interactions were found in the other subgroups (Supplementary Figure S2).

### 3.6 Sensitivity analysis

In the sensitivity analysis, we included the primary diagnosis of patients with tracheal intubation as a covariate in the Cox regression model for analysis. It was found that when NPAR was used as a categorical variable and the second group of NPAR (2.41 ≤ NPAR < 3.38) was used as the reference, in the fully adjusted model, patients with higher levels of NPAR (NPAR≥3.38) had a 60% higher risk of in-hospital mortality compared with the NPAR reference group (2.41 ≤ NPAR < 3.38), and a 61% higher risk of ICU mortality compared with the NPAR reference group (2.41 ≤ NPAR < 3.38) (fully adjusted model, HR range 1.6–1.61, *p* < 0.05) (Supplementary Table S2). This indicates that the relationship between NPAR and in-hospital and ICU mortality remains reliable. We further conducted subgroup analysis based on the primary diagnosis and found no significant interaction (Supplementary Table S3). When we incorporated mechanical ventilation time, ARDS, glucocorticoid use, albumin dosage, and creatinine as covariates into the Cox regression model, the results showed: with NPAR as a categorical variable and using the median group as reference, patients in the high NPAR group still exhibited a 69% increased risk of in-hospital mortality and a 67% increased risk of ICU mortality in the fully adjusted model (fully adjusted model, HR range 1.67–1.69, *p* < 0.05 for all) (Supplementary Table S4).

### 3.7 Incremental predictive value of NPAR over SOFA score

To further evaluate whether NPAR provides incremental predictive value beyond the established ICU severity score, we compared the performance of models with and without NPAR using the NRI and IDI. As shown in Supplementary Table S5, adding NPAR to the SOFA score resulted in a continuous NRI of 0.125 (95% CI: 0.034–0.177, *p* = 0.036). The IDI was 0.002 (*p* = 0.18), and the median improvement in risk score was 0.003 (*p* = 0.024).

## 4 Discussion

Our retrospective study reveals that elevated NPAR (>2.2) in intubated ICU patients associates with increased in-hospital and ICU mortality, demonstrating a U-shaped relationship. The inflection point for mortality risk in this cohort was approximately 2.2, identified through two-piecewise regression analysis. However, this threshold is derived from exploratory data-driven analyses and requires external validation in independent cohorts before its potential clinical significance can be considered.

The biological plausibility of NPAR as a prognostic marker in intubated ICU patients is supported by its integration of two interconnected pathophysiological processes: excessive inflammatory activation and depletion of nutritional metabolic reserves. Neutrophils, as key effectors of the innate immune system, are rapidly recruited to sites of infection during inflammation, where their activation releases reactive oxygen species and chemokines that can lead to oxidative stress and endothelial dysfunction (28–32). Albumin levels, by contrast, primarily reflect nutritional status and colloid osmotic pressure, with serum concentrations inversely correlated with systemic inflammation (33). Low albumin levels, often resulting from malnutrition and inflammation, are well-established prognostic indicators across various diseases (34). In the specific context of intubated ICU patients, tracheal intubation and mechanical ventilation may further amplify this inflammatory-nutritional imbalance. Experimental studies have shown that these procedures alter gene expression related to anaerobic bacteria and biofilms, potentially causing lower respiratory tract dysbiosis and predisposing patients to infections (1, 35). Endotracheal intubation and mechanical ventilation can also directly damage lung tissue through cellular injury and mediator release from activated cells (10).

The prognostic value of NPAR observed in our study is consistent with a growing body of evidence supporting the utility of inflammation-based biomarkers in critically ill populations. Nonlinear relationships between NPAR and clinical outcomes have been reported across diverse diseases, including Kawasaki disease (36) and hypertension (37). Unlike traditional inflammatory markers such as C-reactive protein and procalcitonin, NPAR integrates both inflammatory and nutritional status, potentially offering an advantage in predicting long-term prognosis. As a novel biomarker combining neutrophil percentage and albumin levels, NPAR reflects systemic infection and inflammation (26, 38). A retrospective study from China found that NPAR may become a more accurate and reliable biomarker for predicting spontaneous bacterial peritonitis (39). Another study indicates that elevated NPAR values are associated with an increased 28-day mortality rate in Chinese patients with severe sepsis (40). In critical care settings, elevated NPAR has been independently associated with all-cause mortality in patients with acute kidney injury, myocardial infarction, and cardiogenic shock (17, 41, 42). In the field of oncology, NPAR has also demonstrated its predictive potential to a certain extent. The study by Ko et al. (43), indicates that preoperative NPAR is a strong indicator of the invasiveness of oral squamous cell carcinoma. Another multicenter study points out that NPAR shows to be a clinically significant indicator for predicting worse oncological outcomes in patients with muscle-invasive bladder cancer treated with neoadjuvant chemotherapy and radical cystectomy (44). Other composite inflammatory markers, such as the neutrophil-to-lymphocyte ratio (NLR) and monocyte-to-lymphocyte ratio (MLR), have also shown prognostic value in conditions like acute myocardial infarction (45). However, the predictive performance of these markers may vary by clinical context; for instance, NPAR was not the optimal predictor for all-cause mortality in community-based heart failure patients compared to NLR and platelet-to-lymphocyte ratio (46). This discrepancy underscores the importance of selecting biomarkers based on specific patient characteristics and clinical scenarios.

An interesting observation in our study was the U-shaped relationship, with increased mortality risk at both low and high NPAR values. Below the inflection point of 2.2, lower NPAR values were associated with reduced mortality risk, likely reflecting a well-preserved nutritional status and controlled inflammatory response. However, extremely low NPAR values, though uncommon in this critically ill cohort, may theoretically indicate an immunocompromised state with impaired host defense mechanisms. The clinical implications of low NPAR in intubated patients warrant further investigation in larger studies with greater statistical power to explore this subpopulation.

It should be noted that when NPAR was analyzed as a continuous variable, it was significantly associated with mortality in the crude model; however, this association was no longer statistically significant after full adjustment for confounders, suggesting that it may be influenced by other clinical factors. Nevertheless, when NPAR was analyzed as a categorical variable, higher levels (≥3.38) remained significantly associated with increased mortality risk across all adjusted models, indicating that its prognostic value may be more pronounced at extreme values. This suggests that NPAR should be considered in conjunction with other clinical parameters rather than used in isolation.

In summary, NPAR has demonstrated strong prognostic value across multiple diseases. However, it should be interpreted as a prognostic marker reflecting illness severity and inflammatory burden, rather than a modifiable causal factor, as its components are influenced by multiple interrelated physiological processes. This study is the first to extend this association to the ICU tracheal intubation population and to demonstrate its incremental predictive value over the SOFA score. It should be emphasized that NPAR is not intended to replace established ICU severity scores such as SOFA or APACHE, which integrate multiple organ function parameters. Given its significant improvement in risk reclassification and clinical accessibility, NPAR may serve as a simple and rapid adjunctive tool for initial risk stratification. It is particularly valuable in settings where timely calculation of comprehensive scores is not feasible, serving as a complement to existing scoring systems.

While this study provides valuable insights, we also recognize several limitations. First, this study was conducted using data from a single-center U.S. database (MIMIC-IV). Clinical practices, ICU admission criteria, and patient populations may vary across different regions and healthcare systems, which could limit the generalizability of our findings to other settings. As a single-center retrospective study, it may be subject to selection bias and information bias. It is particularly noteworthy that missing data on endotracheal intubation indications, duration of endotracheal intubation, nutritional indicators, and certain ventilator parameters (such as tidal volume, PEEP, etc.) may introduce residual confounding. Although NPAR was calculated using laboratory values from the first day of ICU admission, whether the measurements were obtained before or after intubation could not be determined. If blood samples were collected after intubation, NPAR values may reflect post-intubation inflammatory effects rather than the baseline status at ICU admission. Additionally, the timing of covariate collection relative to intubation was also unclear, potentially introducing time-dependent confounding. Furthermore, due to missing components of established ICU severity scores in the database, we were unable to calculate these scores and include them in the comparative analysis, which represents another data-related limitation of this study. Although we have strictly controlled for known confounding factors through multivariate regression analysis, the potential influence of unmeasured variables on the study results cannot be completely ruled out. Second, our study was limited to ICU patients with tracheal intubation; whether these findings apply to non-ICU intubated patients requires further investigation. Despite sensitivity analyses adjusting for primary diagnosis, we could not perform subgroup analyses by intubation indication (e.g., surgical vs. medical, elective vs. emergent) or primary etiology (e.g., ARDS vs. neurological) due to database limitations, restricting the applicability of our findings across diverse ICU intubation scenarios. Finally, the observational study design cannot establish a causal relationship between NPAR and outcomes, and all these limitations should be further explored through prospective, multicenter studies.

## 5 Conclusion

Our study suggests that higher NPAR levels may be associated with increased risks of in-hospital and ICU mortality in intubated patients. A non-linear relationship was observed, with an inflection point at approximately 2.2 in this cohort. However, given the single-center retrospective design, these findings require validation in multicenter prospective studies before clinical application.

## Data Availability

Publicly available datasets were analyzed in this study. This data can be found at: https://mimic.physionet.org/ (Reference number: 10.13026/C2X).

## Footnotes

1
https://mimic.physionet.org/

## References

[1] Xue-Meng C Gao-Wang L Xiao-Mei L Fan-Fang Z Jin-Fang X. Effect of mechanical ventilation under intubation on respiratory tract change of bacterial count and alteration of bacterial flora. Exp Lung Res. (2023) 49:165–77. doi: 10.1080/01902148.2023.2264947, 37789686

[2] Menon R Vasani SS Widdicombe NJ Lipman J. Laryngeal injury following endotracheal intubation: Have you considered reflux? Anaesth Intensive Care. (2023) 51:14–9. doi: 10.1177/0310057X221102472, 36168788

[3] Sony S Krishnamurthy J Reddy KN Motiani P Shekhar S. Comparison of normal saline and alkalinized 2% lignocaine to reduce emergence phenomenon and post-intubation morbidities: a prospective, double-blind, randomized study. Cureus. (2023) 15:e33910. doi: 10.7759/cureus.33910, 36819305 PMC9937631

[4] Bauer J Brüggmann D Klingelhöfer D Maier W Schwettmann L Weiss DJ . Access to intensive care in 14 European countries: a spatial analysis of intensive care need and capacity in the light of COVID-19. Intensive Care Med. (2020) 46:2026–34. doi: 10.1007/s00134-020-06229-6, 32886208 PMC7472675

[5] De Jong A Molinari N Terzi N Mongardon N Arnal J-M Guitton C Early identification of patients at risk for difficult intubation in the intensive care unit: development and validation of the MACOCHA score in a multicenter cohort study. Am J Respir Crit Care Med (2013);187:832–839. doi: 10.1164/rccm.201210-1851OC, 23348979 23348979

[6] Casey JD Janz DR Russell DW Vonderhaar DJ Joffe AM Dischert KM . Bag-mask ventilation during tracheal intubation of critically ill adults. N Engl J Med. (2019) 380:811–21. doi: 10.1056/NEJMoa1812405, 30779528 PMC6423976

[7] Amaya S Murillo M Gutiérrez Pérez ML Cervera HS Andrade MJ Zuñiga MA . The role of local inflammation in complications associated with intubation in pediatric patients: a narrative review. Pediatr Anesth. (2023) 33:427–34. doi: 10.1111/pan.14643, 36719267

[8] Al Saeg AA Alnori H. Laryngeal injury and dysphonia after endotracheal intubation. J Med Life. (2021) 14:355–60. doi: 10.25122/jml-2020-0148, 34377201 PMC8321602

[9] Nasrolahzadeh S Nourian J Khosravi A Ghasempour S Abbasi A Ebrahimi H. Comparison of the effect of pressure control and volume control ventilation on endotracheal tube cuff pressure in patients undergoing general anesthesia and mechanical ventilation: a parallel randomized clinical trial. BMC Anesthesiol. (2023) 23:300. doi: 10.1186/s12871-023-02263-1, 37670235 PMC10478180

[10] Dries DJ. Mechanical ventilation: history and harm. Air Med J. (2016) 35:12–5. doi: 10.1016/j.amj.2015.10.006, 26856653

[11] Liu C-F Chien L-W. Predictive role of neutrophil-percentage-to-albumin ratio (NPAR) in nonalcoholic fatty liver disease and advanced liver fibrosis in nondiabetic US adults: evidence from NHANES 2017–2018. Nutrients. (2023) 15:1892. doi: 10.3390/nu15081892, 37111111 PMC10141547

[12] Wang X Zhang Y Wang Y Liu J Xu X Liu J . The neutrophil percentage-to-albumin ratio is associated with all-cause mortality in patients with chronic heart failure. BMC Cardiovasc Disord. (2023) 23:568. doi: 10.1186/s12872-023-03472-9, 37980510 PMC10657562

[13] Lan C Su W Yang M Chen S Wu Y. Predictive role of neutrophil-percentage-to-albumin, neutrophil-to-lymphocyte and eosinophil-to-lymphocyte ratios for mortality in patients with COPD: evidence from NHANES 2011–2018. Respirology. (2023) 28:1136–46. doi: 10.1111/resp.14589, 37655985

[14] Chen Z Xie D Li Y Dai Z Xiang S Chen Z . Neutrophil albumin ratio is associated with all-cause mortality in stroke patients: a retrospective database study. IJGM. (2022) 15:1–9. doi: 10.2147/IJGM.S323114, 35018109 PMC8742575

[15] Sun T Shen H Guo Q Yang J Zhai G Zhang J . Association between neutrophil percentage-to-albumin ratio and all-cause mortality in critically ill patients with coronary artery disease. Biomed Res Int. (2020) 2020:8137576. doi: 10.1155/2020/8137576 32934964 PMC7479485

[16] Gong Y Li D Cheng B Ying B Wang B. Increased neutrophil percentage-to-albumin ratio is associated with all-cause mortality in patients with severe sepsis or septic shock. Epidemiol Infect. (2020) 148:e87. doi: 10.1017/S0950268820000771, 32238212 PMC7189348

[17] Wang B Li D Cheng B Ying B Gong Y Cavalcanti Rolla V. The neutrophil percentage-to-albumin ratio is associated with all-cause mortality in critically ill patients with acute kidney injury. Biomed Res Int. (2020) 2020:5687672. doi: 10.1155/2020/5687672, 32219136 PMC7049452

[18] Xu M Huan J Zhu L Xu J Song K. The neutrophil percentage-to-albumin ratio is an independent risk factor for poor prognosis in peritoneal dialysis patients. Ren Fail. (2024) 46:2294149. doi: 10.1080/0886022X.2023.2294149, 38178381 PMC10773631

[19] Jiao S Zhou J Feng Z Huang J Chen L Li Z . The role of neutrophil percentage to albumin ratio in predicting 1-year mortality in elderly patients with hip fracture and external validation. Front Immunol. (2023) 14:1223464. doi: 10.3389/fimmu.2023.1223464, 37622119 PMC10445888

[20] Zhang H Wu T Tian X Lyu P Wang J Cao Y. High neutrophil percentage-to-albumin ratio can predict occurrence of stroke-associated infection. Front Neurol. (2021) 12:705790. doi: 10.3389/fneur.2021.705790, 34566849 PMC8455847

[21] Zawiah M Khan AH Farha RA Usman A Al-Ashwal FY Akkaif MA. Assessing the predictive value of neutrophil percentage to albumin ratio for ICU admission in ischemic stroke patients. Front Neurol. (2024) 15:1322971. doi: 10.3389/fneur.2024.1322971, 38361641 PMC10868651

[22] El-Benna J Hurtado-Nedelec M Marzaioli V Marie JC Gougerot-Pocidalo MA Dang PM. Priming of the neutrophil respiratory burst: role in host defense and inflammation. Immunol Rev. (2016) 273:180–93. doi: 10.1111/imr.12447, 27558335

[23] Trebicka J Garcia-Tsao G. Controversies regarding albumin therapy in cirrhosis. Hepatology. (2025) 81:288–303. doi: 10.1097/HEP.0000000000000521, 37540192 PMC11643133

[24] Johnson AEW Bulgarelli L Shen L Gayles A Shammout A Horng S . MIMIC-IV, a freely accessible electronic health record dataset. Sci Data. (2023) 10:1. doi: 10.1038/s41597-022-01899-x, 36596836 PMC9810617

[25] Yang Q Zheng J Chen W Chen X Wen D Chen W . Association between preadmission metformin use and outcomes in intensive care unit patients with Sepsis and type 2 diabetes: a cohort study. Front Med. (2021) 8:640785. doi: 10.3389/fmed.2021.640785, 33855034 PMC8039324

[26] Tawfik B Mokdad AA Patel PM Li HC Huerta S. The neutrophil to albumin ratio as a predictor of pathological complete response in rectal cancer patients following neoadjuvant chemoradiation. Anti-Cancer Drugs. (2016) 27:879–83. doi: 10.1097/CAD.0000000000000411, 27434664

[27] Ruan Z Lu T Chen Y Yuan M Yu H Liu R . Association between psoriasis and nonalcoholic fatty liver disease among outpatient US adults. JAMA Dermatol. (2022) 158:745–53. doi: 10.1001/jamadermatol.2022.1609, 35612851 PMC9134040

[28] Döring Y Libby P Soehnlein O. Neutrophil extracellular traps participate in cardiovascular diseases: recent experimental and clinical insights. Circ Res. (2020) 126:1228–41. doi: 10.1161/CIRCRESAHA.120.315931, 32324499 PMC7185047

[29] Honda T Uehara T Matsumoto G Arai S Sugano M. Neutrophil left shift and white blood cell count as markers of bacterial infection. Clin Chim Acta. (2016) 457:46–53. doi: 10.1016/j.cca.2016.03.017, 27034055

[30] Swirski FK Nahrendorf M. Leukocyte behavior in atherosclerosis, myocardial infarction, and heart failure. Science. (2013) 339:161–6. doi: 10.1126/science.1230719, 23307733 PMC3891792

[31] Soehnlein O Libby P. Targeting inflammation in atherosclerosis—from experimental insights to the clinic. Nat Rev Drug Discov. (2021) 20:589–610. doi: 10.1038/s41573-021-00198-1, 33976384 PMC8112476

[32] Vulesevic B Sirois MG Allen BG De Denus S White M. Subclinical inflammation in heart failure: a neutrophil perspective. Can J Cardiol. (2018) 34:717–25. doi: 10.1016/j.cjca.2018.01.018, 29801737

[33] Don BR Kaysen G. Serum albumin: relationship to inflammation and nutrition. Semin Dial. (2004) 17:432–7. doi: 10.1111/j.0894-0959.2004.17603.x 15660573

[34] Goldwasser P Feldman J. Association of serum albumin and mortality risk. J Clin Epidemiol. (1997) 50:693–703. doi: 10.1016/S0895-4356(97)00015-2, 9250267

[35] Bassis CM Erb-Downward JR Dickson RP Freeman CM Schmidt TM Young VB . Analysis of the upper respiratory tract microbiotas as the source of the lung and gastric microbiotas in healthy individuals. MBio. (2015) 6:e00037. doi: 10.1128/mBio.00037-15, 25736890 PMC4358017

[36] Deng L Wang T Duan Y Liu B Jiang J Liu D . Neutrophil percentage-to-albumin ratio is a potential marker of intravenous immunoglobulin resistance in Kawasaki disease. Sci Rep. (2024) 14:15232. doi: 10.1038/s41598-024-66135-5, 38956281 PMC11219825

[37] Liu Z Dong L Shen G Sun Y Liu Y Mei J . Associations of neutrophil-percentage-to-albumin ratio level with all-cause mortality and cardiovascular disease-cause mortality among patients with hypertension: evidence from NHANES 1999–2010. Front Cardiovasc Med. (2024) 11:1397422. doi: 10.3389/fcvm.2024.1397422, 39087072 PMC11288876

[38] Yu Y Zhong Z Yang W Yu J Li J Guo X . Neutrophil percentage-to-albumin ratio and risk of mortality in patients on peritoneal Dialysis. JIR. (2023) 16:6271–81. doi: 10.2147/JIR.S437256, 38146321 PMC10749557

[39] Mousa N Salah M Elbaz S Elmetwalli A Elhammady A Abdelkader E . Neutrophil percentage-to-albumin ratio is a new diagnostic marker for spontaneous bacterial peritonitis: a prospective multicenter study. Gut Pathog. (2024) 16:18. doi: 10.1186/s13099-024-00610-2, 38561807 PMC10985869

[40] Hu C He Y Li J Zhang C Hu Q Li W . Association between neutrophil percentage-to-albumin ratio and 28-day mortality in Chinese patients with sepsis. J Int Med Res. (2023) 51:03000605231178512. doi: 10.1177/03000605231178512, 37314249 PMC10291015

[41] Lin Y Lin Y Yue J Zou Q. The neutrophil percentage-to-albumin ratio is associated with all-cause mortality in critically ill patients with acute myocardial infarction. BMC Cardiovasc Disord. (2022) 22:115. doi: 10.1186/s12872-022-02559-z, 35300600 PMC8932161

[42] Yu Y Liu Y Ling X Huang R Wang S Min J . The neutrophil percentage-to-albumin ratio as a new predictor of all-cause mortality in patients with cardiogenic shock. Biomed Res Int. (2020) 2020:7458451. doi: 10.1155/2020/7458451 33294452 PMC7714577

[43] Ko C-A Fang K-H Tsai M-S Lee Y-C Lai C-H Hsu C-M . Prognostic value of neutrophil percentage-to-albumin ratio in patients with oral cavity Cancer. Cancer. (2022) 14:4892. doi: 10.3390/cancers14194892, 36230814 PMC9564168

[44] Ferro M Babă D-F Cobelli OD Musi G Lucarelli G Terracciano D . Neutrophil percentage-to-albumin ratio predicts mortality in bladder cancer patients treated with neoadjuvant chemotherapy followed by radical cystectomy. Future Sci OA. (2021) 7:FSO709. doi: 10.2144/fsoa-2021-0008, 34258022 PMC8256323

[45] Wu C-C Wu C-H Lee C-H Cheng C-I. Association between neutrophil percentage-to-albumin ratio (NPAR), neutrophil-to-lymphocyte ratio (NLR), platelet-to-lymphocyte ratio (PLR) and long-term mortality in community-dwelling adults with heart failure: evidence from US NHANES 2005–2016. BMC Cardiovasc Disord. (2023) 23:312. doi: 10.1186/s12872-023-03316-6, 37344786 PMC10286403

[46] Dai K Li Z Luo Y Xiong Q Xiong Y Song Z . Neutrophil percentage-to-albumin ratio and monocyte-to-lymphocyte ratio as predictors of free-wall rupture in patients with acute myocardial infarction. J Clin Lab Anal. (2022) 36:e24136. doi: 10.1002/jcla.24136, 34820903 PMC8761430

