# Supplementary material for: Association between the neutrophil-to-albumin ratio and mortality in patients undergoing tracheal intubation: a retrospective cohort study
Source: Front Med (Lausanne). 2026 Apr 1;13:1804074. doi: 10.3389/fmed.2026.1804074 (PMC13079267; doi:10.3389/fmed.2026.1804074)
Supplement: Supplementary file 1 [file Table_1.docx]

**TABLE S1** | Univariate analysis of risk factors for in-hospital mortality and ICU mortality in tracheal intubation.

| **Variables** | In-hospital mortality | | ICU mortality | |
| --- | --- | --- | --- | --- |
|  | **HR(95%CI)** | ***P-*value** | **HR(95%CI)** | ***P-*value** |
| **Age(years)** | 1.03 (1.02,1.03) | < 0.001 | 1.03 (1.02,1.03) | < 0.001 |
| **Gender,(Male vs Female)** | 0.91 (0.75,1.11) | 0.364 | 0.96 (0.76,1.21) | 0.723 |
| **Race/Ethnicity** |  |  |  |  |
| White | Ref |  | Ref |  |
| African Americans | 0.72 (0.49,1.04) | 0.083 | 0.69 (0.44,1.07) | 0.098 |
| Other | 1.13 (0.91,1.41) | 0.268 | 1.08 (0.84,1.4) | 0.559 |
| **Heart rate** | 1.0043 (1.0001,1.0084) | 0.044 | 1.0065 (1.0017,1.0112) | 0.007 |
| **SBP** | 0.9912 (0.987,0.9955) | < 0.001 | 0.99 (0.98,0.99) | < 0.001 |
| **Resp** | 1.02 (1.01,1.04) | < 0.001 | 1.03 (1.02,1.05) | < 0.001 |
| **Spo_2_** | 0.88 (0.81,0.95) | 0.001 | 0.88 (0.8,0.96) | 0.004 |
| **Glucose** | 1.01 (1,1.03) | 0.011 | 1.02 (1.01,1.03) | 0.001 |
| **PT** | 1.01 (1.01,1.02) | < 0.001 | 1.01 (1.01,1.02) | < 0.001 |
| **INR** | 1.13 (1.09,1.17) | < 0.001 | 1.13 (1.09,1.18) | < 0.001 |
| **Na** | 0.98 (0.96,0.99) | 0.005 | 0.98 (0.96,1) | 0.104 |
| **K** | 1.16 (1.07,1.27) | < 0.001 | 1.15 (1.03,1.27) | 0.009 |
| **Ca** | 1.16 (0.87,1.56) | 0.312 | 1.14 (0.81,1.61) | 0.442 |
| **Cl** | 0.98 (0.97,0.99) | 0.004 | 0.98 (0.97,1) | 0.02 |
| **AKI stage** |  |  |  |  |
| 0 | Ref |  | Ref |  |
| 1 | 2.23 (1.75,2.84) | < 0.001 | 3.04 (2.26,4.08) | < 0.001 |
| 2 | 2.35 (1.71,3.22) | < 0.001 | 3.08 (2.11,4.49) | < 0.001 |
| 3 | 3 (2.27,3.96) | < 0.001 | 4.13 (2.97,5.75) | < 0.001 |
| **MI** | 1.47 (1.14,1.91) | 0.003 | 1.44 (1.06,1.95) | 0.02 |
| **COPD** | 1.0001 (0.8023,1.2466) | 0.999 | 1.07 (0.83,1.38) | 0.589 |
| **CVD** | 1.21 (0.95,1.56) | 0.129 | 0.98 (0.71,1.33) | 0.879 |
| **PVD** | 1.27 (0.94,1.72) | 0.113 | 1.03 (0.7,1.5) | 0.888 |
| **Sepsis** | 1.27 (0.96,1.69) | 0.098 | 1.35 (0.96,1.89) | 0.087 |
| **MV** | 0.88 (0.7,1.09) | 0.231 | 1.17 (0.89,1.54) | 0.254 |
| **Elective surgery** | 0.47 (0.07,3.35) | 0.453 | 0.65 (0.09,4.66) | 0.672 |
| **NPAR** | 1.05 (1.02,1.07) | < 0.001 | 1.04 (1,1.07) | 0.025 |

**Abbreviations:** SBP, systolic blood pressure; Resp, respiratory; Spo_2,_ pulse oximetry derived oxygen saturation; PT, prothrombin time; INR, international normalized ratio; Na, sodium; K, potassium; Ca, calcium; Cl, chlorine; AKI stage, acute kidney injury stage; MI, myocardial infarction; COPD, chronic obstructive pulmonary disease; CVD, cerebrovascular disease; PVD, peripheral vascular disease; MV, mechanical ventilation; NPAR, neutrophil percentage-to-albumin ratio.

**TABLE S2** | Relationships between NPAR, in-hospital mortality, and ICU mortality in different models.

| **Variable** | **Crude** | | **Adjust** | |
| --- | --- | --- | --- | --- |
|  | **HR (95% CI)** | ***P-*value** | **HR (95% CI)** | ***P-*value** |
| **Hospital mortality** |  |  |  |  |
| NPAR as continuous | 1.05 (1.02~1.07) | 0.001 | 1.01 (0.98~1.04) | 0.475 |
| 2.41 ≤ NPAR <3.38 | **Ref** |  | **Ref** |  |
| NPAR <2.41 | 0.92 (0.71~1.19) | 0.524 | 1.08 (0.82~1.42) | 0.571 |
| NPAR ≥3.38 | 1.58 (1.25~1.99) | <0.001 | 1.6 (1.24~2.05) | <0.001 |
| P for trend |  | <0.001 |  | <0.001 |
| **ICU mortality** |  |  |  |  |
| NPAR as continuous | 1.04 (1~1.07) | 0.025 | 0.99 (0.96~1.03) | 0.7 |
| 2.41 ≤ NPAR <3.38 | **Ref** |  | **Ref** |  |
| NPAR <2.41 | 0.95 (0.7~1.29) | 0.738 | 1.17 (0.85~1.6) | 0.345 |
| NPAR ≥3.38 | 1.59 (1.21~2.09) | 0.001 | 1.61 (1.2~2.17) | 0.002 |
| P for trend |  | <0.001 |  | 0.002 |

**Notes:** Crude model was not adjusted.

Adjust model adjust for gender + age + MI + COPD + CVD + PVD + Sepsis + race + heart rate + SBP + Resp + Spo_2_ + glucose + PT + INR + Na + K + Cl + AKI stage + Ca + MV + Elective surgery + First diagnosis.

**Abbreviations:** MI, myocardial infarction; COPD, chronic obstructive pulmonary disease; CVD, cerebrovascular disease; PVD, peripheral vascular disease; SBP, systolic blood pressure; Resp, respiratory; Spo_2,_ pulse oximetry derived oxygen saturation; PT, prothrombin time; INR, international normalized ratio; Na, sodium; K, potassium; Cl, chlorine; AKI stage, acute kidney injury stage; Ca, calcium; MV, mechanical ventilation.


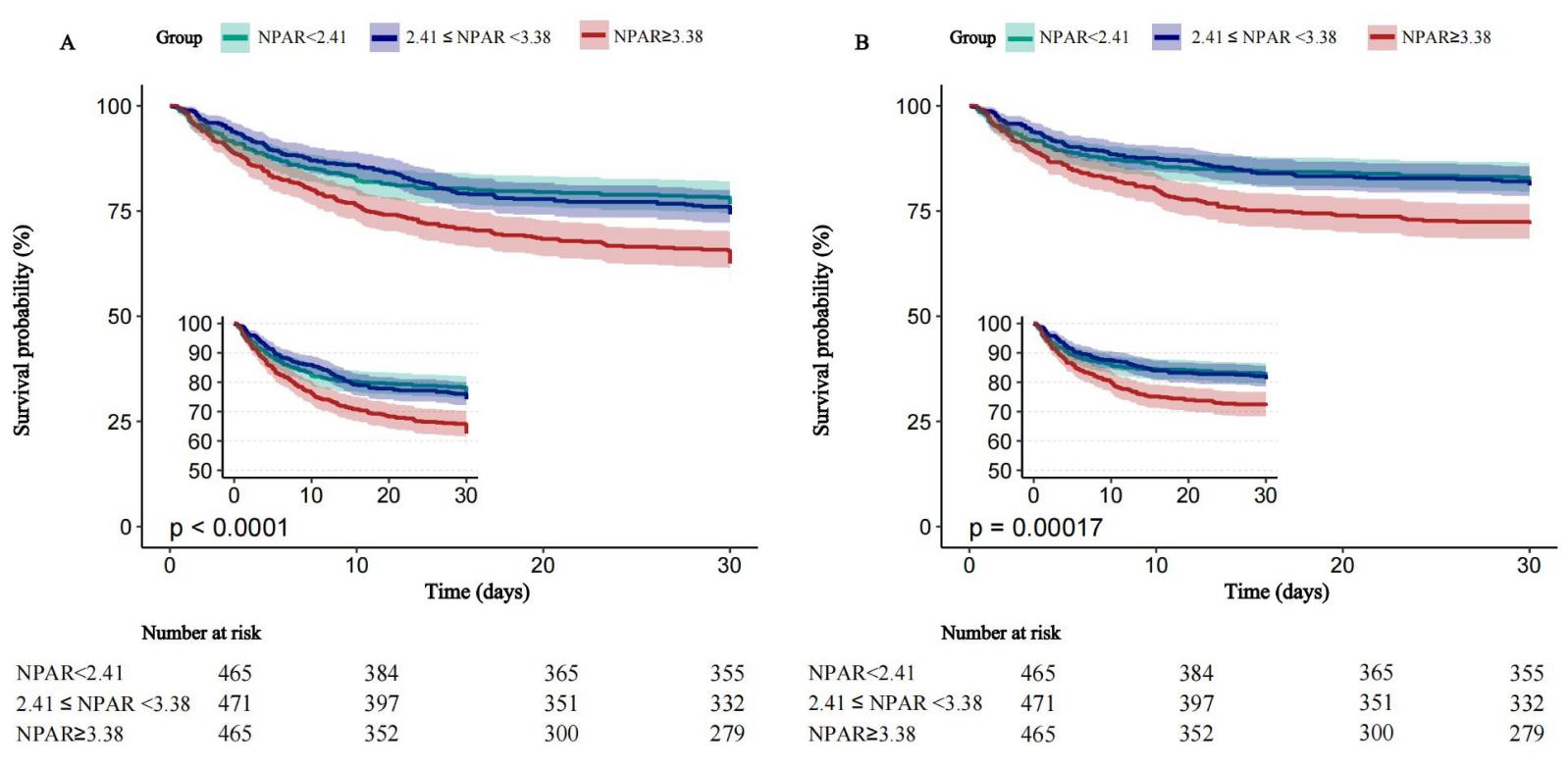


**Figure S1. **A**** Kaplan-Meier survival analysis of TyG and ICU mortality in patients with tracheal intubation. ****B**** Kaplan-Meier survival analysis of TyG and in-hospital mortality in patients with tracheal intubation.


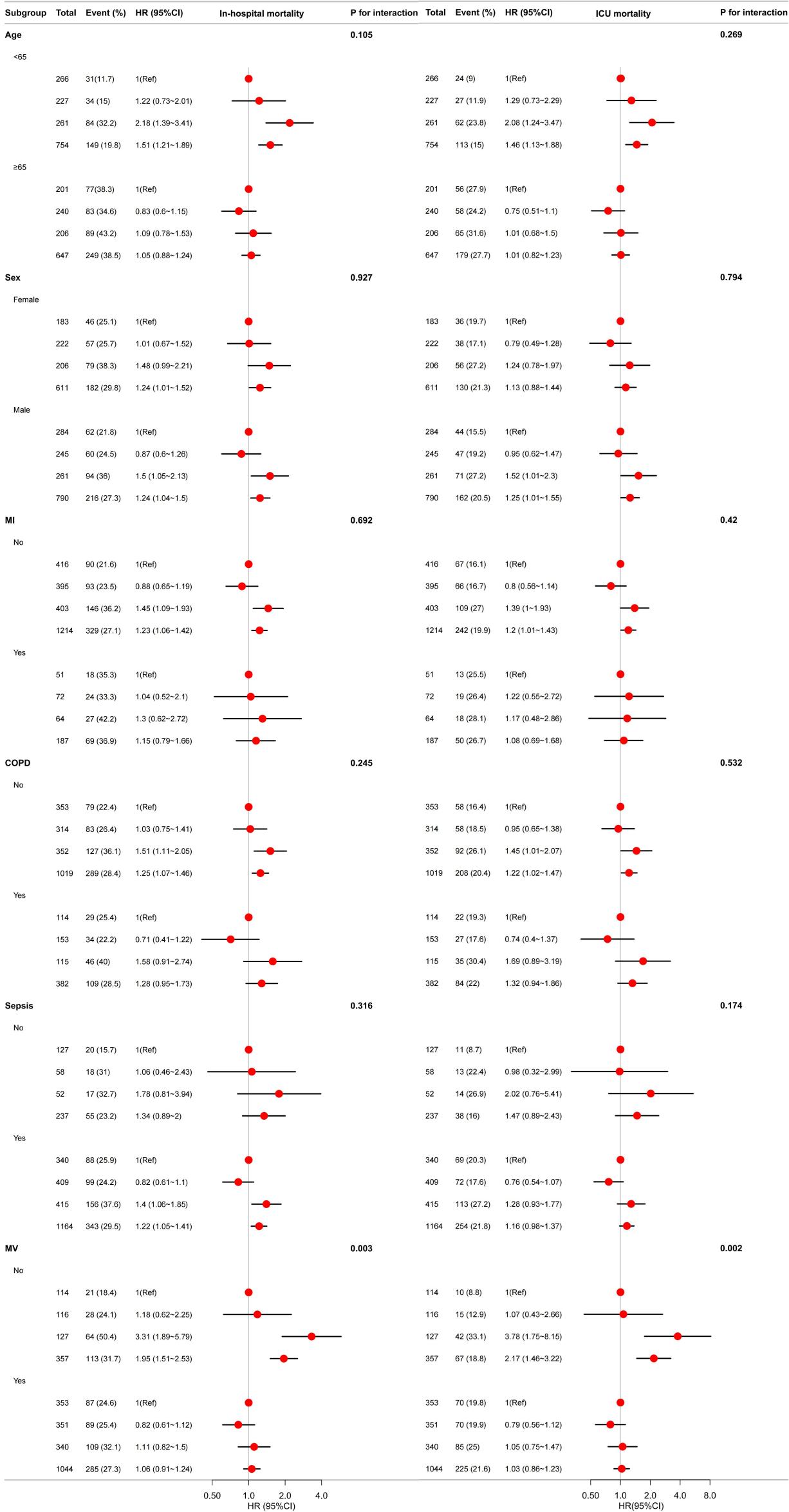


**Figure S2.** Subgroup analysis of the association between NPAR and in-hospital mortality and ICU mortality.

**TABLE S3** | Subgroup analysis of the association between NPAR and in-hospital mortality and ICU mortality.

| **Subgroup** | **N** | **Hospital mortality** | | | **ICU mortality** | | |
| --- | --- | --- | --- | --- | --- | --- | --- |
|  |  | **HR(95% CI)** | ***P*-value** | ***P* for interaction** | **HR(95% CI)** | ***P*-value** | ***P* for interaction** |
| **First diagnosis** |  |  |  | 0.333 |  |  | 0.151 |
| **Neurological Disorders** | 216 | 0.93 (0.73~1.19) | 0.585 |  | 1.04 (0.75~1.44) | 0.807 |  |
| **Respiratory System Diseases** | 206 | 1.28 (0.99~1.66) | 0.064 |  | 1.37 (1.01~1.86) | 0.043 |  |
| **Cardiovascular System Diseases** | 139 | 0.94 (0.68~1.29) | 0.687 |  | 1 (0.73~1.38) | 0.982 |  |
| **Digestive System Diseases** | 204 | 1.16 (1.02~1.3) | 0.018 |  | 1.08 (0.94~1.24) | 0.281 |  |
| **Infectious Diseases** | 331 | 1 (0.96~1.05) | 0.964 |  | 0.96 (0.89~1.03) | 0.232 |  |
| **Others** | 304 | 0.96 (0.84~1.09) | 0.549 |  | 0.96 (0.8~1.16) | 0.692 |  |

Adjusted for gender + age + MI + COPD + CVD + PVD + Sepsis + race + heart rate + SBP + Resp + Spo_2_ + glucose + PT + INR + Na + K + Cl + AKI stage + Ca + MV + Elective surgery + First diagnosis.

**TABLE S4** | Relationships between NPAR, in-hospital mortality, and ICU mortality in different models.

| **Variable** | **Crude** | | **Adjust** | |
| --- | --- | --- | --- | --- |
|  | **HR (95% CI)** | ***P-*value** | **HR (95% CI)** | ***P-*value** |
| **Hospital mortality** |  |  |  |  |
| NPAR as continuous | 1.05 (1.02~1.07) | 0.001 | 1.01 (0.98~1.04) | 0.527 |
| 2.41 ≤ NPAR <3.38 | **Ref** |  | **Ref** |  |
| NPAR <2.41 | 0.92 (0.71~1.2) | 0.539 | 1.14 (0.86~1.49) | 0.362 |
| NPAR ≥3.38 | 1.6 (1.26~2.02) | <0.001 | 1.69 (1.31~2.17) | <0.001 |
| P for trend |  | <0.001 |  | <0.001 |
| **ICU mortality** |  |  |  |  |
| NPAR as continuous | 1.04 (1~1.07) | 0.025 | 0.99 (0.96~1.03) | 0.642 |
| 2.41 ≤ NPAR <3.38 | **Ref** |  | **Ref** |  |
| NPAR <2.41 | 0.95 (0.7~1.29) | 0.727 | 1.21 (0.88~1.66) | 0.246 |
| NPAR ≥3.38 | 1.6 (1.21~2.1) | 0.001 | 1.67 (1.24~2.24) | 0.001 |
| P for trend |  | 0.001 |  | 0.001 |

**Notes:** Crude model was not adjusted.

Adjust model adjust for gender + age + MI + COPD + CVD + PVD + Sepsis + race + heart rate + SBP + Resp + Spo_2_ + glucose + PT + INR + Na + K + Cl + AKI stage + Ca + MV + Elective surgery + First diagnosis + creatinine + mechanical ventilation time + ARDS + glucocorticoid + albumin dosage.

**Abbreviations:** MI, myocardial infarction; COPD, chronic obstructive pulmonary disease; CVD, cerebrovascular disease; PVD, peripheral vascular disease; SBP, systolic blood pressure; Resp, respiratory; Spo_2,_ pulse oximetry derived oxygen saturation; PT, prothrombin time; INR, international normalized ratio; Na, sodium; K, potassium; Cl, chlorine; AKI stage, acute kidney injury stage; Ca, calcium; MV, mechanical ventilation; ARDS, Acute Respiratory Distress Syndrome.
